# Retraction: Medical Data Feature Learning Based on Probability and Depth Learning Mining: Model Development and Validation

**DOI:** 10.2196/98673

**Published:** 2026-04-28

**Authors:** JMIR Publications Editorial Office

**Affiliations:** 1 JMIR Publications Toronto, ON

The JMIR Publications Editorial Office is retracting the article:

Medical Data Feature Learning Based on Probability and Depth Learning Mining: Model Development and Validation [1]

This action is being taken due to identified concerns regarding potential manipulation of the submission process, the integrity of the peer review process, and the relevance of several references.

The concerns with this publication were identified during an investigation of a series of articles with related concerns. The authors were contacted and offered an opportunity to comment on these findings and the proposed retraction but did not respond to any attempts at communication.
